# Effectiveness of using 2D atlas and 3D PDF as a teaching tool in anatomy lectures in initial learners: a randomized controlled trial in a medical school

**DOI:** 10.1186/s12909-023-04960-4

**Published:** 2023-12-15

**Authors:** Fatma Sıla Eroğlu, Beyza Erkan, Sevil Buket Koyuncu, Zeynep Rumeysa Komşal, Feray Ekin Çiçek, Müşerref Ülker, Merve Ebrar Toklu, Melike Atlan, Yavuz Selim Kıyak, Serdar Kula, Özlem Coşkun, Işıl İrem Budakoğlu

**Affiliations:** 1 Gazi University Faculty of Medicine, Ankara, Turkey; 2 Department of Medical Education and Informatics, Gazi University Faculty of Medicine, Gazi Üniversitesi Hastanesi E Blok 9. Kat, Beşevler, Ankara, 06500 Turkey; 3 Department of Pediatrics, Gazi University Faculty of Medicine, Ankara, Turkey

**Keywords:** Anatomy education, 3D PDF, Anatomy atlas, Gross anatomy, Liver anatomy, Genitalia anatomy, Video lecture, Medical students, Health professions education, Experimental study

## Abstract

**Background:**

Anatomy is a crucial part of medical education, and there have been attempts to improve this field by utilizing various methods. With the advancement of technology, three-dimensional (3D) materials have gained popularity and become a matter of debate about their effectiveness compared to two-dimensional (2D) sources. This research aims to analyze the effectiveness of 3D PDFs compared to 2D atlases.

**Methods:**

This study is a randomized controlled trial involving 87 Year-1 and Year-2 medical students at Gazi University Faculty of Medicine, Turkey. The study was conducted in two steps. In Step-1, students were randomized to watch lecture videos on liver anatomy and male genitalia anatomy supplemented with either a 3D PDF (intervention group) or 2D atlas (control group) images. Following the video lectures, a test (immediate test) was administered. In Step-2, the same test (delayed test) was administered 10 days after the immediate test. The test scores were compared between the intervention and control groups. In addition to the descriptive analyses, Chi-square and Mann-Whitney U tests were performed.

**Results:**

In the immediate test, while there was no significant difference between the groups for the liver test (*p* > 0.05), 3D PDF group’s scores (Median = 24.50) was significantly higher than the 2D atlas group’s in the genitalia test (Median = 21.00), (*p* = 0.017). The effect size (Cohen’s d) was 0.57. In the delayed test, there was no significant difference between the groups in the liver and genitalia tests (*p* > 0.05). However, the effect size in the immediate genitalia test was 0.40. Year-1 students’ immediate test of genitalia performances were significantly higher in the 3D PDF group (Median = 24.00) than the 2D atlas group (Median = 19.00), (*p* = 0.016). The effect size was 0.76. Also, Year-1 students’ 3D PDF group (Median = 20.50) presented with significantly higher performance than the 2D atlas group (Median = 12.00), (*p* = 0.044) in the delayed test of genitalia, with the 0.63 effect size.

**Conclusion:**

3D PDF is more effective than 2D atlases in teaching anatomy, especially to initial learners. It is particularly useful for teaching complex anatomical structures, such as male genitalia, compared to the liver. Hence, it may be a valuable tool for medical teachers to utilize during lectures.

## Introduction

Medical education requires a holistic approach to the human body. Learning anatomy is one of the most essential elements in the preclinical education process of medical students [1]. Understanding complex spatial components of the body is necessary to integrate theoretical knowledge into clinical practices, such as physical examination and arriving at a diagnosis [2].

Throughout the years, various methods have been utilized for anatomy education. Traditional approaches included cadaver dissections and physical models, which provided a three-dimensional (3D) perspective. Additionally, two-dimensional (2D) materials such as atlases, chalk drawings, and slide presentations have played a significant role in facilitating the study outside of the laboratory. However, since these materials are limited in providing stereoscopic sight, they were highly dependent on an individual’s cognitive abilities to visualize the structures [3]. Hence, anatomy remained one of the most challenging subjects to conceptualize in medical education [4–6].

Over time, due to reduction of laboratory hours [7], difficulties in obtaining cadavers [8], and personal challenges faced by students, novel types of learning opportunities became necessary. The advancement of technology has led to the use of three-dimensional digital tools, such as 3D static images, 3D animations, and applications, which allow users to manipulate the structures. Thus, these developments raised new questions about the effectiveness of these various methods.

The literature on this topic demonstrates variations in the effectiveness of these methods when compared to one another. While some have compared physical models to computer-based 3D anatomy programs and found that 3D digital models are less effective [6, 9], many studies focused on comparing 2D sources and 3D models, including virtual and augmented reality, and found that 3Ds are more effective in general [10–12]. However, there are still controversies on the effectiveness of these methods. In critical appraisal of the existing literature, we found several factors behind these controversies. It may be because these sources and models have a wide range of definitions causing confusion among researchers [13], and each study focuses on a different material. The other dissimilarities between the studies are participant backgrounds (medical students, residents, nursing students), anatomical structures they used (e.g. female pelvis [14], hepatobiliary system [2], heart [15]), and study design (self-learning, classroom settings) [10, 12]. This heterogeneity also may explain the controversies in the literature.

The utilization of these models, whether for self-learning by students or as teaching materials by instructors, may also have contributed to the emergence of disputed results in the literature. The majority of studies focused on students’ self-learning experiences. Nevertheless, according to Mayer’s cognitive theory of multimedia, the optimal way of learning for students is by using both images and narration in an electronic learning environment [16]. This can be interpreted as highlighting the importance of the initial learning with the help of an instructor followed by the student’s studies.

Traditionally, teachers use slide presentations with atlas images and provide narration in lecture videos. Since 3D models were proposed as promising tools in many studies [10, 12], they may also be a prominent way to enhance learning in the lectures. However, availability as well is crucial for sustainability. For instance, when the studies are critically appraised, it can be seen that the cost and inaccessibility of new technologies, such as virtual reality, may be a barrier especially in lower income settings [12]. Since 3D PDFs are more affordable and accessible than virtual reality [17] and can be read on most modern digital devices [18], they may be a practical substitute for 2D atlases in lectures. However, there is limited research on the comparison of these materials in lectures and measuring the effectiveness on initial learning. Moreover, the effect of different complexity levels of anatomical structures on learning through these materials is unclear.

In this randomized controlled experiment, we sought to investigate the effect of using 3D PDFs instead of 2D atlases on learning in lectures. The research question was “Is 3D PDF more effective than 2D atlas for using in the lectures as a visual material to teach complex and less complex anatomical structures to initial learners?”.

We chose two different anatomical structures by considering their complexity to test the hypothesis that 3D PDF would be more effective than traditional atlas for teaching both complex and less complex anatomical structures. In order to investigate its effect on initial learning, the lecture videos were presented to medical students who had never studied the structures before. Subsequently, short and long-term retention performances were tested. The results of this study may shed light on the effectiveness of 3D PDF, which is a free and more accessible tool to teach anatomy.

## Methods

### Trial design

We followed the CONSORT statement [19] in reporting this randomized, controlled, parallel-group study that was approved by the Gazi University Institutional Review Board (code: 2022 − 1073). Since “single-group pretest–posttest designs suffer from many validity threats” and “pretest often weaken the study design” [20], we opted to use randomized controlled experimental design without a pretest .

We evaluated the effectiveness of 3D PDFs and traditional 2D atlases in teaching two topics: Liver anatomy and male genitalia anatomy. These structures were specifically selected based on their different levels of anatomical complexity, with male genitalia anatomy being considered more complex in comparison to liver anatomy. The complexity was determined based on consensus among the research group that consists of professors, medical educationists, medical doctors, and medical students.

**Fig. 1:**
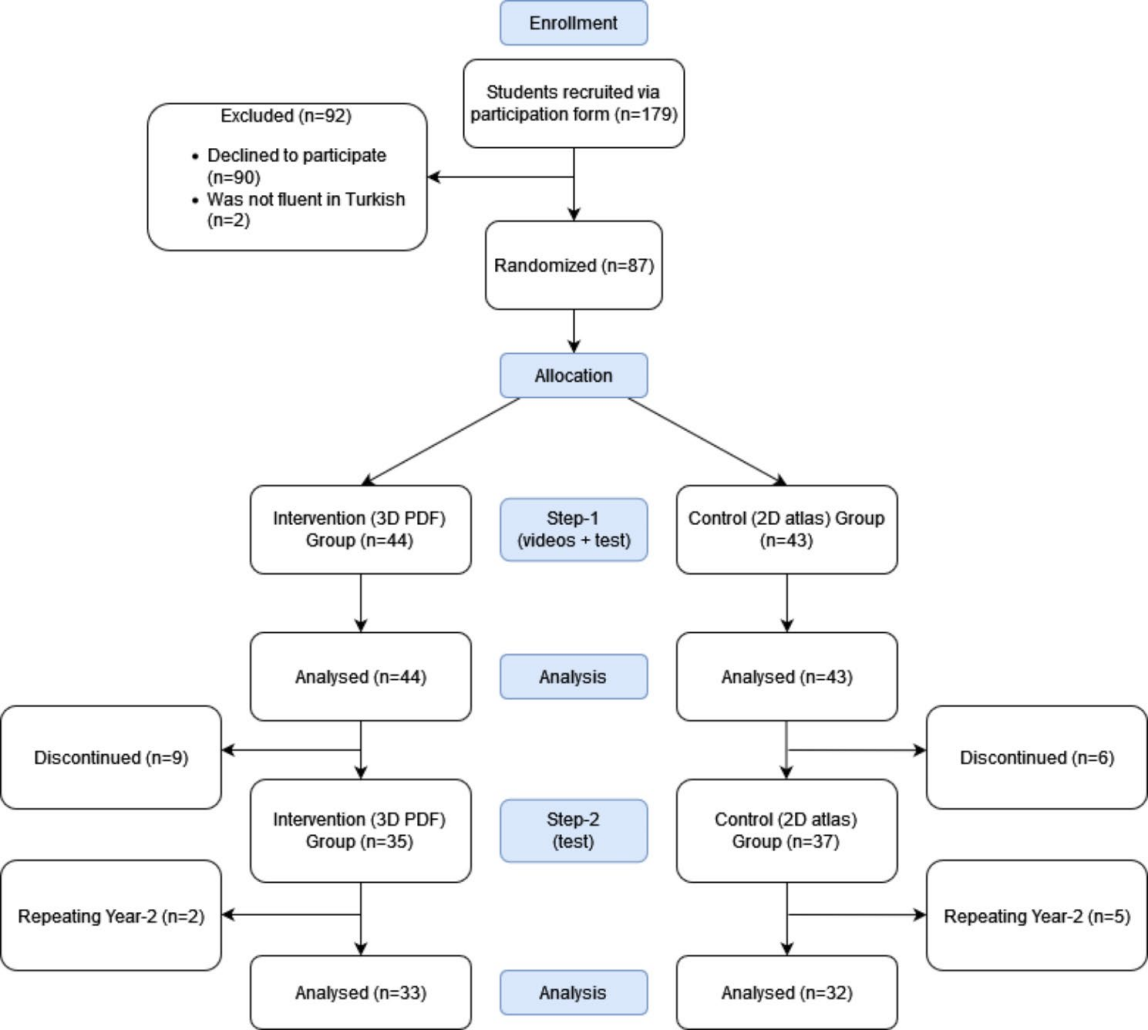
Trial process

The study consisted of two steps. In Step-1, students were randomly assigned to either the intervention group (who watched lecture videos included 3D PDFs) or the control group (who watched lecture videos included 2D atlas images). After the video lectures, a test (the immediate test) was administered to assess their retention performance. In Step-2, the same test (the delayed test) was administered 10 days after the immediate test. Figure 1 presents the trial process.

### Participants

The study included Year-1 and Year-2 medical students in 2022–2023 term at Gazi University Faculty of Medicine, Ankara, Turkey. All Year-1 (n = 571) and Year-2 (n = 488) medical students were invited to the study through messaging groups covered the entire population. They had not yet taken anatomy classes on liver anatomy and male genitalia anatomy within the curriculum. Moreover, Year-1 students did not take any anatomy lecture yet. Therefore, the intervention served for them as a form of pure initial learning. It should also be noted that some of the Year-2 students were repeating their second year of study, which meant they had previously taken anatomy classes on the chosen topics. However, this did not pose any threat to the validity of the study since the grouping was randomized in Step-1 and their scores were excluded from the analysis in Step-2. Considering previous research and a power analysis table [21], a minimum sample size of 35 participants per group was calculated to achieve a power of 0.80, alpha of 0.05, and effect size of 0.70.

The participation to the study was voluntary, and all participants provided informed consent. Recruitment was carried out via a participation form shared with all Year-1 and Year-2 medical students, resulting in a total of 179 students being recruited. Of these students, 87 participated in Step-1, and 72 out of the 87 students completed Step-2, as presented in Fig. 1.

### Materials

Educational materials included lecture videos on liver anatomy and male genitalia anatomy. The liver anatomy videos covered liver topography, gallbladder, and biliary system. The male genitalia anatomy videos covered male internal and external genitalia.

Four videos (two for each topic) using the same written transcripts in each pair but different visual materials (images from Netter Anatomy Atlas [22] for the control group, and 3D PDFs of liver [23] and male genitalia [24] by Visible Korean [accessible from http://vkh3.kisti.re.kr/?q=node/12, the zipped “PDF file (Male).pdf” file in the page] for the intervention group) were recorded on each topic. Medical students recorded the videos based on written transcripts, by articulating the content word for word. The transcripts were prepared by the research team that comprised of not only medical students but also medical doctors, specialists, and medical educationists. The videos were in Turkish and were the same length, 5 min for liver anatomy and 10 min for male genitalia anatomy. The resolution of the videos was the same. The only difference between the videos was the visual material (3D PDF vs. 2D atlas). The lecturer explained the anatomical structures by simultaneously hovering the cursor over the mentioned structures. The lecturer manipulated the models of the structures to show them from different angles and perspectives in 3D PDF videos.

To assess the retention performance of the students, a written test with answer keys was developed for each topic based on the video content by the research team which comprised of medical students, medical doctors, specialists, and medical educationists who are experienced in assessment. The tests were piloted by medical students who had not participated in the test development process. After revisions based on feedback, the liver anatomy test had 10 questions worth a total of 20 points, while the male genitalia anatomy test had 19 questions worth a total of 40 points. Both tests contained open-ended and multiple-choice questions, and did not include any visual material.

### Trial process

The participants were randomly assigned to two groups (3D PDF and 2D atlas). Simple randomization was performed using SPSS v22.0 for Windows, ensuring that each participant had an equal chance of being assigned to either group.

The trial took place at Gazi University Faculty of Medicine. The videos were displayed to the participants in a classroom setting through a projector. Two proctors were present in each classroom, and identical procedures were followed in all classrooms throughout the trial. At the beginning of the trial, the purpose of the study was explained to the participants, and they were instructed not to engage in any distracting or cheating behavior.

In Step-1, all groups underwent the same process, including watching the liver videos twice (5 min + 5 min), completing the liver test (10 min), watching the genitalia videos twice (10 min + 10 min), and completing the genitalia test (20 min). The same videos were watched two times by considering that they had no prior learning on these topics and watching only once would lead to a cognitive overload. They were provided with printed versions of the video transcripts and were allowed to take notes while watching the videos. These paper prints were collected prior to administering each test. Step-1 lasted for one hour.

In Step-2, the delayed test, which is identical to the immediate test, was administered 10 days after the completion of Step-1 to evaluate the difference in performance between the groups in the long-term. Multiple sessions were arranged to accommodate the availability of the participants, and they were instructed not to share information about the test with other participants between sessions to ensure the reliability of the study. They had not been informed that the tests will be identical. The delayed test was carried out in the same classroom settings with proctors.

Both the immediate and delayed tests were administered as written exams (paper and pencil test), and their scoring involved at least two researchers using the answer key. If there were any disagreements in scoring, they were resolved with the help of the third researcher.

### Statistical analysis

Statistical analyses were carried out using SPSS v22.0 for Windows. A *p*-value < 0.05 was considered statistically significant.

In order to ensure that randomization worked well, the group characteristics in terms of gender, year, and repeating Year-2 were compared between the intervention group and the control group by using Chi-squared test.

The Step-1 liver anatomy and male genitalia anatomy (immediate test) scores were compared between the groups to evaluate the difference in short-term retention. Since the data violated normality assumptions, Mann-Whitney U test was performed to investigate whether there was a significant difference in retention performance between the two groups.

The same comparison was carried out in Step-2 (delayed test) for evaluating long-term retention. For the validity of the analysis, participants who were repeating Year-2 were excluded from the Step-2 analysis because they were not re-randomized into the groups. Due to the violation of normality assumptions, Mann-Whitney U test were performed to compare the scores of the two groups.

Based on the fact that our participants comprised of Year-1 and Year-2 medical students, we conducted subgroup analyses (Mann-Whitney U test) both in the immediate test and the delayed test scores.

To evaluate the reliability of the tests, Cronbach’s alpha values were calculated. The acceptable level of Cronbach’s alpha values was determined as 0.70 [25]. Item analysis was conducted using Microsoft Excel to calculate item difficulty (by dividing the total score of test-takers by the maximum score of test-takers can get) and item discrimination (“proportion of high achievers (top 27% in terms of total score) who answered the item correctly” minus “proportion of low achievers (bottom 27% in terms of total score) who answered the item correctly”) indices.

Effect sizes were calculated using Cohen’s d, and were interpreted as small, medium, and large for values of 0.2, 0.5, and 0.8, respectively [26].

## Results

### Participants

A total of 87 participants volunteered for the experiment. They were randomized into two groups: 3D PDF and 2D atlas. Group characteristics are shown in Table 1.

The 3D PDF (intervention) group had a female majority (65.9%) and the 2D atlas (control) group had again a female majority (58.1%). The two groups did not significantly differ in terms of gender, study year, and repeating Year-2 (*p* > 0.05).

87 participants took the immediate tests on liver and male genitalia immediately after watching each video about liver and male genitalia anatomy.

**Table 1:** Participant Characteristics

		Immediate Test			**Delayed Test**		
		Participants (n)		p-value*	Participants (n)		p-value*
		Atlas	3D PDF		Atlas	3D PDF	
**Gender**							
	*Female*	25	29	0.455	19	23	0.384
	*Male*	18	15		13	10	
**Year of Study**							
	*1st -Year*	24	22	0.587	21	20	0.675
	*2nd -Year*	19	22		11	13	
**Repeating Year-2**		5	6	0.778	0	0	-

*Chi-squared test

### Immediate test

There was no significant difference between the performances of the 3D PDF group (Median = 15.00) and the 2D atlas group (Median = 17.00) in the liver test, U = 900.00, Z = -0.39, *p* = 0.695. In the genitalia test, however, the 3D PDF group (Median = 24.50) performed significantly higher than the 2D atlas group (Median = 21.00), U = 665.50, Z = -2.38, *p* = 0.017. The effect size (Cohen’s d) in the genitalia test was 0.57. The results of both the intervention and control groups’ test results are presented in Table 2.

Among Year-2 medical students, neither in the liver test (*p* > 0.05) nor in the genitalia test (*p* > 0.05), the performances did not significantly differ between the 2D atlas group and the 3D PDF group. Although Year-1 medical students’ liver test performances were not significantly different between the 2D atlas group (Median = 13.00) and the 3D PDF group (Median = 14.00), *p* = 0,860, the genitalia test performances among Year-1 students were significantly higher in 3D PDF group (Median = 24.00) than the 2D atlas group (Median = 19.00), U = 155.00, Z = -2.40, *p* = 0.016. The effect size was 0.76.

**Table 2:** Statistical analysis of immediate and delayed tests

		Mean (Standard Deviation)	Median	***p***-value*	Cohen’s d
**Immediate Test**					
Liver					
	*Atlas*	15.00 (4.10)	17.00	0.695	0.13
	*3D PDF*	14.41 (4.74)	15.00		
Male Genitalia					
	*Atlas*	20.42 (8.02)	21.00	0.017	0.57
	*3D PDF*	24.59 (6.29)	24.50		
**Delayed Test**					
Liver					
	*Atlas*	10.16 (5.28)	9.50	0.519	0.10
	*3D PDF*	10.70 (4.70)	12.00		
Male Genitalia					
	*Atlas*	16.25 (7.54)	18.50	0.118	0.40
	*3D PDF*	19.18 (6.93)	21.00		

*Mann-Whitney U test

### Delayed test

In the liver test, there was no significant difference between the performances of the 3D PDF group (Median = 12.00) and the 2D atlas group (Median = 9.50), U = 479.00, Z = -0.64, *p* = 0.519. In the genitalia test, similarly, there was no significant difference between the 3D PDF group (Median = 21.00) and the 2D atlas group (Median = 18.50), U = 409.00, Z = -1.56, *p* = 0.118. However, the effect size in the genitalia test was 0.40.

Year-1 students’ liver test performances did not differ significantly between the 2D atlas group (Median = 9.00) and the 3D PDF group (Median = 10.00), *p* = 0.794. However, Year-1 medical students’ genitalia test performances were significantly higher in the 3D PDF group (Median = 20.50) than 2D atlas group (Median = 12.00), U = 133.00, Z = -2.01, *p* = 0.044. The effect size was 0.63. Year-2 medical students’ performances were not significantly different between the two groups, neither in the liver test (*p* > 0.05) nor in the genitalia test (*p* > 0.05).

### Item analysis

Item analysis was performed on both the immediate and delayed tests by calculating item difficulty and discrimination indices. In the immediate test, the mean difficulty and discrimination indices of 10 items in the liver test were 0.72 and 0.52, respectively, while they were 0.56 and 0.47 in the genitalia test. In the delayed test, the mean difficulty and discrimination indices of 19 items in the genitalia test were 0.51 and 0.43, respectively, while they were 0.42 and 0.62 in the liver test, respectively.

In the immediate test, Cronbach’s alpha value was 0.70 in the liver test, and 0.78 in the genitalia test. In the delayed test, the value was 0.69 in the liver test, and 0.72 in the genitalia test. All of the values are above of or close to the acceptable level, which is 0.70.

## Discussion

Our study aimed to compare the effects of 3D PDF (intervention) and 2D atlas (control) on teaching anatomic structures with different levels of complexity in video lectures to initial learners. Their effects on short-term and long-term retention were assessed.

While there were non-significant differences with little effect sizes between the intervention group and the control group in the liver tests (neither in the immediate nor in the delayed), medium effect sizes showed superiority of 3D PDF over 2D atlas in the immediate and the delayed genitalia tests. The effect sizes, which were 0.76 in the immediate test and 0.63 in the delayed test, were close to be classified as a large effect (0.80) [26] when only the pure initial learners, who are Year-1 medical students with no prior knowledge to anatomy, are considered.

Our results demonstrated that 3D PDF is superior to 2D atlas for teaching the anatomy of male genitalia, which is a more complex structure, yet not for teaching the liver anatomy, which is a less complex structure, both in terms of short-term retention and long-term retention. Therefore, it indicated that if the complexity of the anatomical structures is high, teaching with 3D PDFs provides a better environment for initial learners than teaching with 2D anatomy atlases.

A previous research [27] compared the effect of 3D PDF and printed images of cardiac anatomy on nursing students’ performance and found that 3D PDF provides similar levels of benefit in the short-term compared to printed images, but 3D PDF required significantly lower levels of cognitive effort, which are similar levels of effort that autostereoscopic holograms required. Similarly, another study showed that learning through a 3D application enabled students to spend less time to complete assignments compared to 2D atlas [28]. Even if the experiments included self-study rather than lectures, they may help us to explain the superiority of 3D PDF from the perspective of cognitive load theory [29]. It can be interpreted in a way that 2D atlas required more cognitive effort compared to 3D PDF, and therefore it led to a cognitive overload in the complex structure, not in the less complex one. For this reason, 3D PDF group outperformed 2D atlas group in the genitalia test, while the performance in the liver test was similar. This is supported by a study that demonstrated that the cognitive workload required to learn pelvis anatomy, a structure with low level of complexity as liver is, is similar when utilizing computer-generated 3D models or static images [30].

3D PDFs offer considerable practical benefits over 2D atlases in anatomy education, even though they are relatively recent material in anatomy education, not widely used by either teachers or students. Our findings suggest that 3D PDFs may be a favorable choice for teaching complex anatomical concepts, as stereopsis has a critical role in learning anatomy [31]. It may have potential applications for teaching complex anatomical concepts rather than using 2D atlases since its relative novelty can draw students’ attention and motivate them to participate more actively in anatomy classes. Concordantly, several studies showed that students are in favor of using 3D PDFs and 3D computer models, not only in health professions education settings [2, 28, 32, 33] but also in other science education contexts [34]. Moreover, since students and teachers are familiar with PDF readers as a file reading tool, extraneous cognitive load [29] that arises from adopting a new tool would be low in comparison to some other brand new tools. Taking extraneous load into account is essential because it needs to be considered in the development of 3D digital anatomical models [35].

This study has some limitations. First, it is a single-center study in a country with its unique context, therefore it may not be generalizable. Second, due to the discontinuation of a number of participants and the resulting uneven distribution of students repeating Year-2 between the groups, it was necessary to analyze the long-term scores with a reduced number of participants. While our goal was to minimize participant dropout by maintaining communication through e-messaging groups during the study, dropouts might have led to find a non-significant *p*-value in the delayed genitalia test despite the fact that the effect size was 0.40. Third, the effect size was reported using Cohen’s d although the data violated the normality assumptions, however, it still provides valuable information to interpret the results. Fourth, due to using written assessment, our tests were not sufficient to measure spatial ability of the participants. Future research may evaluate retention performance using the tools such as cadavers or physical models, which allow a more authentic assessment. It is also important to note the limitation that the complexity of the structures was determined by consensus among the research team, not referring to an external source, even though we tried to find an evidence on this classification in the literature. Another limitation was that collecting qualitative data based on student feedback would enhance the ability to better understand and interpret the findings.

## Conclusions

3D PDF is more effective than 2D atlas in teaching anatomy, especially to initial learners with no prior anatomy knowledge. It is particularly useful for teaching complex anatomical structures, such as male genitalia, compared to liver. Therefore, it may be a valuable tool for medical teachers to utilize during lectures in order to prepare medical students for the clinical environment.

## Data Availability

The dataset supporting the conclusions of this article is available in the Zenodo repository, accessible from 10.5281/zenodo.7830028.

## Declarations

### Previous presentations

Preliminary results of this study titled ‘3D PDF: A Promising Learning Tool for Anatomy Education’ has been presented at the AMEE 2023 Conference, August 26–30, 2023, in Glasgow, UK.
